# Hand injury from pneumatic needle gun

**DOI:** 10.11604/pamj.2018.29.46.14638

**Published:** 2018-01-18

**Authors:** Rodolfo Mendes Queiroz, Fred Bernardes Filho

**Affiliations:** 1 Department of Radiology and Imaging, Santa Casa da Misericórdia of Avaré, Avaré, São Paulo, Brazil; 2 CENTROMED Diagnóstico por Imagem, Avaré, São Paulo, Brazil; 3 Dermatology Division, Department of Medical Clinics, Ribeirão Preto Medical School, University of São Paulo, Ribeirão Preto, Brazil

**Keywords:** Hand injuries, occupational risks, occupational health

## Image in medicine

A 37-year-old male complained of pain and swelling of his left hand for 7 days. He was a woodworker and reported that he was using a pneumatic needle gun when the pain began. The patient claimed to have seen a scorpion in the workplace and he believed to have suffered a sting. Initially he sought an emergency room, where hydrocortisone and ketoprofen were administered, and loratadine was prescribed. Because it does not show improvement with seven days of loratadine, he sought the dermatology service. On physical examination, heat, redness and swelling of whole left hand was observed. Dermoscopy with a handheld dermoscope (DermLite II Pro 3Gen) showed a single puncture wound over volar surface of the third left metacarpal. Hand X-ray revealed a foreign body in soft tissue of third left metacarpal. The diagnosis of hand injury from pneumatic needle gun was made. Foreign body was identified and removed. Intravenous antibiotics were administered pre-operatively and oral antibiotics continued post-operatively. Nail gun injuries commonly occur related to improper use by the operator and not following occupational health and safety requirements for operating a nail gun. The amount of energy required to cause serious injury is fairly low: penetration of the skin occurs with projectile velocities of 150 feet per second, whereas bony fractures may occur with projectile velocities of 195 feet per second. Mechanisms of nail gun injury include direct penetration, shrapnel wounds from exploding cartridges and high-pressure injection injuries from the compressed air used to activate the gun. Assessment of a patient with a puncture wound and suspected foreign body begins with a careful history and physical examination.

**Figure 1:**
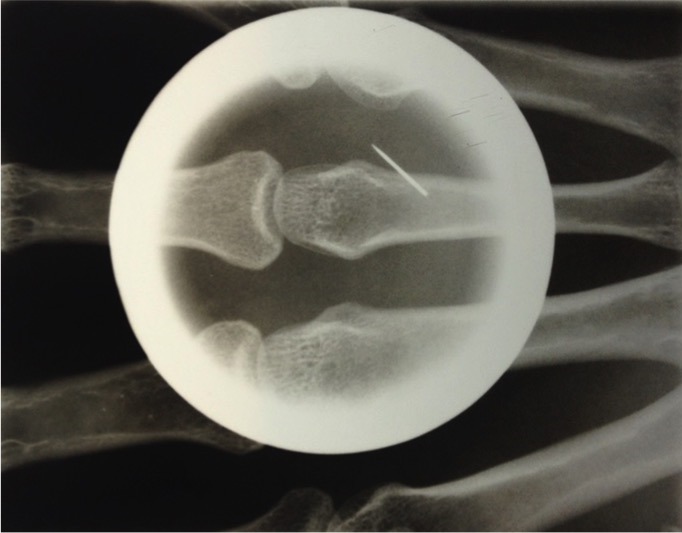
a metal density focus is noted in soft tissue of third left metacarpal; no fracture/dislocation is noted

